# Conserving diversity in Irish plant–pollinator networks

**DOI:** 10.1002/ece3.9347

**Published:** 2022-10-04

**Authors:** Laura Russo, Úna Fitzpatrick, Michelle Larkin, Sarah Mullen, Eileen Power, Dara Stanley, Cian White, Aoife O'Rourke, Jane C. Stout

**Affiliations:** ^1^ Department of Ecology and Evolutionary Biology University of Tennessee Knoxville Tennessee USA; ^2^ Botany Department Trinity College Dublin Dublin 2 Ireland; ^3^ National Biodiversity Data Centre Waterford Ireland; ^4^ Botany and Plant Science, School of Natural Sciences Ryan Institute, National University of Ireland Galway Galway Ireland; ^5^ School of Agriculture and Food Science University College Dublin Dublin 4 Ireland

**Keywords:** agricultural intensification, conservation, flower‐visiting insects, grasslands, plant‐pollinator networks

## Abstract

Beneficial insects provide valuable services upon which we rely, including pollination. Pollinator conservation is a global priority, and a significant concern in Ireland, where over half of extant bee species have declined significantly in recent decades. As flower‐visiting insects rely on flowering plants, one way to conserve and promote pollinator populations is to protect high‐quality habitat. We analyzed the structure of insect–flower interactions from multiple habitat categories in a large database of interactions from Ireland. Our primary goals were to compare spatial and temporal variation in Irish network structures, compare Irish networks to published networks from other countries, and provide evidence‐based recommendations for pollinator conservation in Ireland by identifying well‐visited plant species that may promote high pollinator diversity, abundance, and functional complementarity. Habitat types within Ireland differed substantially: seminatural grasslands had the highest pollinator species richness and largest number of unique pollinator species, while intensively managed habitats exhibited negative asymmetry (more plant than pollinator species). This negative asymmetry is notable because most plant–pollinator networks exhibit a positive asymmetry. Within intensively managed habitats, agricultural and urban habitats differed. Urban habitats had the highest number of non‐native plant species while agricultural habitats had the lowest pollinator species richness. We also found Irish networks varied across the growing season, where July had the highest plant and insect species richness. When comparing Irish networks to published networks from other countries, we found Irish networks had a higher ratio of plant species to pollinator species, and that this difference was most evident in agricultural habitats. This ratio means the typical network asymmetry (more pollinator than plant species) was flipped (more plant than pollinator species) in the Irish network. We conclude that conserving seminatural grasslands in Ireland will be an essential component of pollinator conservation and identify thirty‐five plant species important for restoring seminatural habitats.

## Graphical abstract

We show that semi‐natural grasslands are critical to pollinator conservation in Ireland. We also show that intensive land management affects the structure of Irish networks, and that urban and agricultural land‐use affect these networks differently.

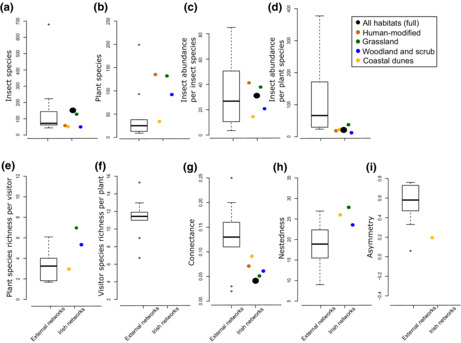

## 1 INTRODUCTION

Globally, ecological systems have been substantially altered by human activities (Ellis & Ramankutty, 2008). For example, the primary effect of agricultural food production at a local scale is the loss of species through the application of agrochemicals and conversion of land toward high‐yield monocultures (Flohre et al., 2011; Grab et al., 2019; Matson et al., 1997; Tscharntke et al., 2005). Other forms of human activities alter ecosystems differently, for example, through the introduction and promotion of non‐native ornamental species, such as in urban or developed landscapes (Godefroid & Koedam, 2007). Regardless of mechanism, habitat degradation has unfavorable implications for the preservation of beneficial biodiversity, particularly those species that provide ecosystem services which maintain sustainable human production systems (Biesmeijer et al., 2006; Hallmann et al., 2017; Lister & Garcia, 2018; Ollerton et al., 2014; Powney et al., 2019).

Complex mutualistic networks of plant–pollinator interactions provide valuable ecosystem services for agricultural production (Dainese et al., 2019; Klein et al., 2007; Rader et al., 2016) alongside their natural role in plant reproduction more broadly (Ollerton et al., 2011). The importance of pollinator diversity for agricultural yield has been demonstrated by many studies (Calderone, 2012; Dainese et al., 2019; Garibaldi et al., 2016). At the same time, agricultural land‐use can have serious negative impacts on pollinator diversity (Grab et al., 2019). The impacts of human activities can be observed in highly coevolved networks of interacting plants and pollinators, for example, by showing increases in non‐native plant and insect taxa over time (Mathiasson & Rehan, 2020). As species in such networks are mutually dependent, species loss can have cascading implications for the persistence of the species with which they interact. Changing environmental quality can also be reflected in structural changes, for example, by altering the asymmetry of insect–flower networks (Soares et al., 2017). Asymmetry is considered a fundamental attribute of mutualistic network structure, and may reflect important coevolutionary patterns (Bascompte et al., 2006), so changes to asymmetry may impact the stability of these interactions over time. The introduction of non‐native species may also increase species richness and add novel interactions in a way that leaves the overall network structure unchanged, while changes in interactions between native species are not detectable at the network level (Lopezaraiza‐Mikel et al., 2007).

It is thus essential to understand and mitigate the impacts of human land‐use on beneficial pollinating insects. It is clear that the highest priority conservation action is protecting remaining intact, high‐quality habitat. A secondary objective is improving the quality of degraded systems, for example, by identifying central organisms in plant–pollinator networks that support both a diversity and abundance of partners. As organisms contribute to community structure on multiple levels, it can be important to evaluate them using various metrics.

To better understand the state of plant–pollinator interactions within Ireland, we addressed three main aims. To understand how habitat quality affects plant–pollinator interactions in Ireland, we first compared networks within Ireland to one another. We compared the structure of networks in intensively managed (agricultural and urban), coastal dune, grassland (seminatural and managed), and woodland/shrubland habitats in Ireland. We further compared changes in these Irish networks across the growing season. Then, to view Irish networks in a broader context, we compared Irish network structures to published networks from other countries. Finally, we evaluated the role of individual species within the Irish networks to provide evidence‐based recommendations for habitat restoration or conservation‐oriented planning.

## 2 MATERIALS AND METHODS

### 2.1 Irish data

We collated data from six different studies in Ireland where obligate flower‐visiting insects (specifically Lepidoptera, bees, and hoverflies) were observed/collected while foraging on flowers from May through August in 2009–2011 and 2017 and April through October in 2018. Sites were selected for surveys based on various research studies and questions, and surveys were conducted by the authors of this study (Larkin & Stanley, 2021; O'Rourke et al., 2014; Power & Stout, 2011; Stanley & Stout, 2013). The average time spent surveying a given transect was 131.10 ±7.92 minutes (range: 2–4108). The average area surveyed among the different surveys was 998.43  ± 19.94 m^2^ (range: 20–5000). Sites were surveyed an average of 38.13 ± 2.83 times (range: 1–15) (Table S1).

The pollinator visitation data were collected by standard hand‐netting transect survey methods aggregated from multiple years of research studies and sites. In most cases, the surveyor also quantified the floral abundance of the different plant species visited during a transect. Floral abundance was quantified as the number of floral units per plant on the transect. We used flower size, as reported by an online database of Irish wildflowers (www.irishwildflowers.ie) to approximate the total floral display (floral abundance × inflorescence size). We used floral area as a measure of floral availability instead of number of inflorescences because inflorescences vary in size among different plant species. In other words, a plant may have many small inflorescences or few large inflorescences and have a similar floral display. For each study, the surveyor identified both insects and plants to the highest resolution possible, and collected insects for later identification in the laboratory when field identification was not possible.

These studies comprised more than 940 h of surveys of plant–pollinator interactions across 709,721 m^2^ and 119 study sites in four broad habitat categories (Table S1): (1) cultivated and built land (agricultural crops [including *Miscanthus*, winter wheat, oil seed rape]), silage and dairy pastures, and urban and suburban gardens and flower beds; (2) coastland (primarily coastal dune systems); (3) grassland and marsh (primarily seminatural and managed grasslands); and (4) woodland and scrub (primarily hedgerows and non‐native shrubs). Our habitat classification was based on the Fossitt (2000) guide to Irish habitats, which has three separate levels of categorization. At the first level, there are 11 habitat types defined within Ireland, from which we sampled four thoroughly. It is worth noting that at the first level of categorization, managed (improved for agriculture) grasslands and seminatural grasslands are grouped together in the broad category of “grassland.” We therefore further separated the habitats into a level 2 classification. There are 30 subcategories of habitat types at level 2, of which we sampled from 18 (Figure S1; Table S1). For the level 2 classification, we were able to separate managed grasslands from seminatural grasslands. We selected the most extensive habitat types that represented the majority of the land surface in Ireland. A rarefaction analysis showed our sample coverage for these four habitats varies from 96.6% (coastland) to 99.3% (cultivated and built land) (Figure S2). When comparing habitat types, we excluded records where the surveyor included more than one habitat type in a single survey.

From these datasets, we automatically excluded any data where the host plant or insect visitor were not resolved to at least genus. Although most specimens were resolved to the species level, we included some specimens that had only been identified to genus, as some plant and insect taxa were difficult/impossible to resolve to species, often listed as “aggregates” (e.g., *Taraxacum agg*. and *Bombus lucorum agg*.). These studies excluded plants that were present but not visited by the target insects.

### 2.2 Statistical software packages

We conducted all of our statistical analyses using R (R Core Team, 2013). To conduct a rarefaction analysis, we used the package *iNEXT* (Chao et al., 2014). To analyze the attributes of the bipartite networks, we used the package *bipartite* (Dormann et al., 2009), specifically calculating the weighted and unweighted degrees of plant species and insect visitor species, network level connectance, nestedness (using NODF), and asymmetry. In addition to the attributes calculated using the *bipartite* package, we conducted a functional complementarity analysis (Devoto et al., 2012) with the package *vegan* (Oksanen, 2011) and calculated centrality by creating a one mode, unweighted projection of plant and insect species in the networks using the package *igraph* (Csardi & Nepusz, 2006). To calculate the effect of floral display size on visitation, we used the package *lme4* (Bates et al., 2014). We also used this package to determine whether visitation to non‐native plants was different than to native plants, given their floral display. NMDS plots of flower‐visitor communities in different habitat types and months of the year were constructed using the package *vegan* (Oksanen, 2011).

### 2.3 Habitat type and phenology in Irish networks

We compared the network structure among four primary habitat types where interactions were recorded in Ireland, intensively managed (agricultural or developed), coastal dunes, grasslands (including both seminatural and managed), and woodlands/shrublands. We quantified the species richness of plants and pollinators in each habitat, along with the number of non‐native plant species. We constructed networks weighted by the abundance of insect visitors on plant species for each habitat type based on the interactions recorded and calculated plant and insect visitor species richness, and weighted and unweighted degree, as well as network connectance, nestedness (NODF), and asymmetry (Table S2). We then identified insect species common to all habitat types (Table S3), as well as those unique to each habitat type in this step (i.e., recorded in only one habitat type) (Table S4). We compared the flower‐visitor communities in these habitats using a nonmetric multidimensional scaling (NMDS) ordination. To determine the relative role of agriculture versus other processes, we further divided habitats into (a) primarily agriculture versus urban intensively managed habitat types, and (b) seminatural versus managed (species‐poor and used for agricultural purposes) grasslands, and evaluated their attributes. These subcategories were assigned according to level two of the Fossitt habitat classifications (Table S1).

Next, using the same data, we constructed separate networks for each of the five months which comprised most of the surveys (May–September). For this comparison, we constructed networks from data pooled across years for each month and reported the same measures calculated above (Table S2). For both habitat and phenology (i.e., month) data, we constructed NMDS plots to visualize any overlap in flower‐visitor community structure.

### 2.4 Null models for specialization metrics

To test the degree of network‐level and species‐level specialization, we compared the existing networks to null network models constructed using the *nullmodel* function in the package *bipartite*. We used this function to generate 1000 null models for each habitat (the full Irish network, human‐modified habitats, coastal dunes, grasslands, and woodlands) and month (the full Irish network, May, June, July, August, and September). Null networks are generated using the *vaznull* method, which randomizes the total number of interactions in the original network, while keeping the connectance constant (Vazquez & Aizen, 2003). We then calculated the mean network‐level specialization (H_2_’), and specialization of the two separate groups of organisms (insects and plants) (d’) across all null models (Blüthgen et al., 2006). We used z‐scores to compare the true values to the null model averages.

### 2.5 Comparing the Irish network to other plant‐visitor networks

To determine whether the attributes of the Irish network were similar to other published flower‐visitor networks, we downloaded publicly available network data from the Interactions Web Database and DataDryad (Table 3). We specifically downloaded networks of quantitative plant–insect visitor interactions with more than a total of 50 species (including both plant and insect species) represented, to ensure the networks were similar in size to our network, as some network attributes are sensitive to networks smaller than 50 species (Dormann et al., 2009). We found nine such networks in the Interaction Web Database and one in DataDryad.

We included island (Kaiser‐Bunbury et al., 2017; Kato et al., 1990; Memmott, 1999), high latitude (Elberling & Olesen, 1999; Magrach et al., 2018; Memmott, 1999), agricultural (Magrach et al., 2018), and recently unglaciated site (Barrett & Helenurm, 1987; Elberling & Olesen, 1999; Magrach et al., 2018; Memmott, 1999; Vázquez & Simberloff, 2002) networks. The downloaded networks varied in sampling effort (36–1525 hours, 1–6 years) and total insect visits recorded (383–12,235) (Table 3). As some of these networks included non‐syrphid dipterans, we repeated the analysis while excluding these groups from the datasets, but saw no significant difference in our results (Figure S6; Table S5).

Using the downloaded datasets, we calculated the species richness of plants and insect visitors, and the following network measures: weighted and unweighted average degrees for plant and insect species, as well as the connectance, nestedness (NODF), and asymmetry of the networks. We selected these measures because they are commonly evaluated in flower–visitor network studies and have been well‐studied, with theoretical underpinnings relating to stability, co‐evolution, and robustness to species loss (Table S2). Moreover, they have been suggested as conservation targets and potentially an important component of monitoring (Tylianakis et al., 2010). As we were comparing a single value (i.e., the value of the Irish network), we determined that the Irish network was significantly different when its value was more than two standard deviations from the mean of the other published networks.

### 2.6 Ranking Irish plant and insect species

There tends to be an inherent background relationship between flower‐visitor abundance and the size of the floral display (Russo et al., 2013). Flower‐visitors may exhibit a preference for plant species by visiting them in greater abundance than expected based on their availability, described here as the size of the floral display. In contrast, a plant species with a large floral display but low visitor abundance may be considered less preferred. Therefore, using generalized linear mixed effects models (GLMMs), we tested the relationship between floral area and the abundance and richness of insect visitors to determine whether more insects were attracted to a larger floral display. We used a log‐log transformation to evaluate the relationship between abundance, richness, and floral area. To account for repeated measures, site and date of sampling were used as random effects in these models. We also investigated whether visitation to a plant family was greater than anticipated given the background abundance and floral display of the species within that family.

The contributions of individual plant and insect species to the structure of the Irish networks were ranked according to several different metrics (Table S2). We categorized these species by functional complementarity (calculated with a hierarchical clustering algorithm) (Devoto et al., 2012), average abundance of visitors (weighted degree) and average partner species richness (unweighted degree), visitation rate (abundance of visitors divided by the floral display), and node longevity (duration of species activity in the network across the weeks of the summer), as well as centrality in unweighted one‐mode projections of the network (closeness and betweenness centrality) (Russo et al., 2013). We normalized these values for all species on a scale of 0–1, where 0 represents minimum values and 1 represents maximum values (i.e., feature scaling), to give different measures equal weight. For plant species, we also created subsets of the network data and recalculated the network parameters separately based on visitation by the following insect subgroups: bees, syrphid flies, and Lepidoptera. Depending on management objectives, it may be preferable to optimize different measures to focus on plant species conservation and restoration. Land managers cannot construct insect communities in the same fashion as plant communities, but this information may be useful for targeting conservation objectives toward more central pollinators, or for protecting key agricultural pollinators.

We used Pearson Correlation Coefficients to test for correlations between the measures listed above. Most of these measures significantly correlated with one another, with the exception of visitation rate, which could be therefore considered an independent measure (Table S6). Functional complementarity was negatively correlated with all of the other measures, and likely also provides a contrasting measure of a species' role in the community.

To test whether non‐native plant species differed significantly from native plant species in terms of their role in the networks, we generated GLMMs containing the following fixed effects: the status of the plant (native or non‐native) and the responses of functional complementarity, weighted and unweighted degree, node longevity, visitation rate, and betweenness and closeness centrality. To correct for multiple testing, we used a Bonferroni correction (alpha = 0.007). For these models, site and week of the year were inserted as random effects to account for repeated measures, and the fixed effects were categorical (native or non‐native species and habitat type). We used a Poisson distribution in these models.

## 3 RESULTS

We analyzed flower‐visitor interactions between 239 flowering plant species and 148 insect visitor species in Ireland (Figure S1). The composition of the insect visitor species (species richness of different visitor groups) observed was: 54.7% hoverflies (Syrphidae), 30.7% bee (Anthophila), and 14.6% butterflies and moths (Lepidoptera). However, bees dominated in terms of abundance on flowers (61% bee, 35% hoverfly, and 4% butterfly or moth visits), similar to other published studies (Rader et al., 2016).

### 3.1 Habitat type and phenology in Irish networks

Although intensively managed habitats had the highest plant species richness, they also had the highest proportion of non‐native plant species. Moreover, they had proportionally lower insect species richness, leading to the most negative asymmetry; intensively managed habitats had 2.44 times as many flowering plant species as insect visitor species (Table 1). The only habitat where the asymmetry was not negative (i.e., more visitor species than plant species) was coastal dune systems, which also had the highest connectance. In wood and shrubland habitats, there remained nearly twice as many plant as insect species (Table 1). The grassland habitats (including both seminatural and managed) had the highest insect species richness overall, along with the greatest average abundance and species richness of insect visitors per plant species. Due to their high plant species richness, and corresponding low insect species richness, intensively managed habitats also had the highest abundance of insects per visitor species, and plant species richness per visitor, along with the highest nestedness (Table 1).

**TABLE 1:** Irish network properties arranged according to the full Irish network (italics) and four main habitat types and their subsets (divided by human land‐use) as well as the proportion of non‐native plant species.

		Four main habitat types				Subsets				Full
		Intensively‐managed	Coastal dune	Grassland	Woodland/Shrubland	Agriculture only	Urban	Seminatural grassland	Amenity grassland	All habitats
Richness	Plant	**134**	33	131	91	56	96	90	79	*238*
	Visitor	55	48	**125**	47	25	42	107	70	*148*
	% Non‐native Plant Richness	**39.6**	3	17.4	39.5	9.3	50	6.6	21.5	*31.7*
Average abundance of visitors (Weighted Degree)	Plant	16.75	20.61	**35.76**	10.54	24.43	8.55	30.6	23.87	*38.39*
	Visitor	**40.82**	14.17	37.48	20.4	56.96	19.55	25.74	26.94	*61.74*
Average species richness of partners (Unweighted Degree)	Plant	3.77	4.21	**6.61**	2.73	4.27	3.02	7.14	4.34	*6.41*
	Visitor	**9.18**	2.9	6.93	5.28	9.56	6.9	6.01	4.9	*10.31*
Connectance		0.07	**0.09**	0.05	0.06	0.17	0.07	0.07	0.06	*0.04*
Nestedness (NODF)		**30.91**	25.86	27.71	23.43	46.22	30.38	27.97	27.9	*30.99*
Asymmetry		−0.42	**0.19**	−0.02	−0.32	−0.38	−0.39	0.09	−0.06	*−0.23*

*Note:* We also include subsets of the main habitat types where they are distinguished by human land‐use. For example, the intensively managed habitats can be divided into agriculture only or urban habitats, while the grasslands can be separated into seminatural or amenity (improved for nonagricultural usage).

The highest value for each measure is indicated in bold.

**FIGURE 1:**
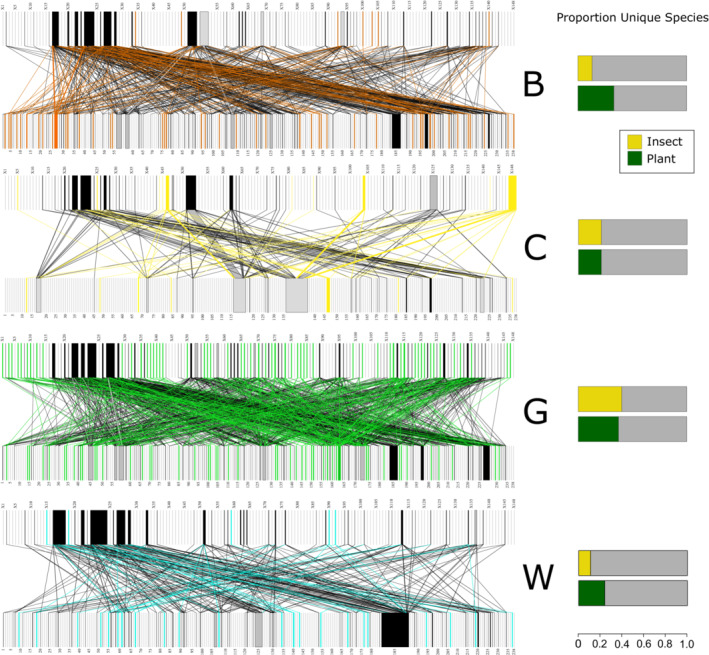
Bipartite networks of interactions between insect visitor species (top row), and flowering plant hosts (bottom row), in the four different primary habitat categories: cultivated and intensively managed land (B), coastal dunes (C), grasslands (G), and woodland and scrub (W). The species unique to each habitat are colored in orange, yellow, green, and blue, respectively. Species found in all habitat types are in black. We also include bar charts of the proportions of unique plant (green) and insect (yellow) species in each habitat type on the right‐hand side.

**FIGURE 2:**
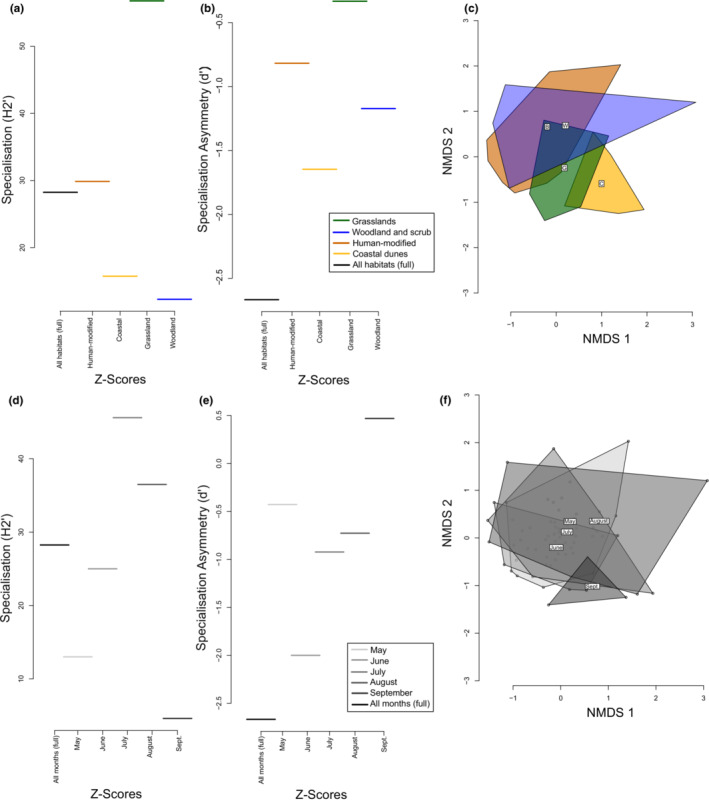
The z‐scores of the full Irish network (black) and habitat subsets as compared to null models for the mean specialization index (H_2_’) of the network of the habitats (a, b) and months (d, e) within the Irish network as compared to null model simulations. The grasslands have the highest overall specialization across the full network and least negative specialization asymmetry (relative to the mean of the null models). NMDS ordination plots of the flower‐visitor communities in the different habitats (c) and months (f).

There was substantial overlap in insect visitor species across habitats; the most abundant visitors were found in all habitats and visited the largest number of plant species (Figure 1, Table S3). On the other hand, only four plant species were found in all four habitat types (*Rubus fruticosus* agg. L.*, Senecio jacobaea* L.*, Taraxacum* agg. L., and *Trifolium repens* L.), each of which has a global distribution, suggesting wide ecological tolerance. The highest proportion of unique insect visitor and plant species was found in grasslands (Table S4), which also had the highest visitor species richness (Figure 1), average visitor abundance (effect size 0.58, *z* value 2.36, *p* = .02), specialization (H_2_’), and specialization asymmetry (d’), relative to the null model comparison (Figure 2a,b). Approximately 40% of both insect and plant species recorded in grasslands were only found in that habitat type (Table S4). The grasslands contrasted with the intensively managed habitats, which had the lowest proportion of unique species (Table S4). The woodland/shrubland habitats had the lowest specialization and the full network had the most negative specialization asymmetry (Figure 2a,b). We also found that woodland/shrubland flower‐visitor communities largely overlapped with the intensively managed flower‐visitor communities, while grasslands and coastal dunes tended to have separate flower‐visitor communities (Figure 2c).

As the proportion of non‐native plant species increased, asymmetry and nestedness tended to decrease across the habitat types (Table 1; Figure S3). However, agricultural habitat types were distinct; they had a low proportion of non‐native plant species, a strongly negative asymmetry, and a high nestedness (Table 1; Figure S3). Grasslands managed for agricultural use through the application of fertilizers and herbicides had a negative asymmetry and high nestedness, and the highest proportion of non‐native species of the habitat subsets (Table 1; Figure S3).

We compared network measures for different months of the flowering season and found that the month of July stood out as having the highest abundance‐related measures and total richness, but that the month of August had a higher average partner species richness and connectance. The highest nestedness and the most positive asymmetry were found in the month of September, although that month also had the lowest species richness overall. The asymmetry was more negative in the full network than in any one phenological subset of the network (Table 2). This was likely driven by the fact that the plant species had a shorter duration of activity during the flowering season (node longevity) than the insect species (plants: 3.72 ± 0.24 weeks of activity vs. insects: 5.45 ± 0.47 [mean ± standard error]). July had the highest specialization (H_2_’) and September had the highest specialization asymmetry (d’), while September had the lowest relative specialization and the full network had the lowest specialization asymmetry compared to a null model (Figure 2d,e). The NMDS ordination plots show significant overlap in flower‐visitor communities among all the months except for September (Figure 2f).

**TABLE 2:** The phenology of network attributes of the full Irish network (italics), and separated by the months of the year where the majority of observations were collected.

		May	June	July	August	Sept	Full
Richness	Plant	74	125	**127**	123	21	*239*
	Visitor	72	93	**95**	86	25	*148*
Average abundance of visitors (Weighted Degree)	Plant	9.86	15.89	**26.6**	23.07	9	*18.99*
	Visitor	10.14	21.35	**35.56**	33	7.56	*30.66*
Average species richness of partners (Unweighted Degree)	Plant	3.49	4.52	4.62	**5.04**	3.48	*6.39*
	Visitor	3.58	6.08	6.18	**7.21**	2.92	*10.32*
Connectance		0.05	0.05	0.05	**0.06**	0.14	*0.04*
Nestedness (NODF)		18.12	25.75	29.02	28.18	**30.96**	*30.99*
Asymmetry		−0.014	−0.15	−0.14	−0.18	**0.09**	*−0.24*

*Note:* Bolded values indicate the highest value for each measure (excluding the full network).

The highest value for each measure is indicated in bold.

### 3.2 Comparing Irish networks to other networks

**FIGURE 3:**
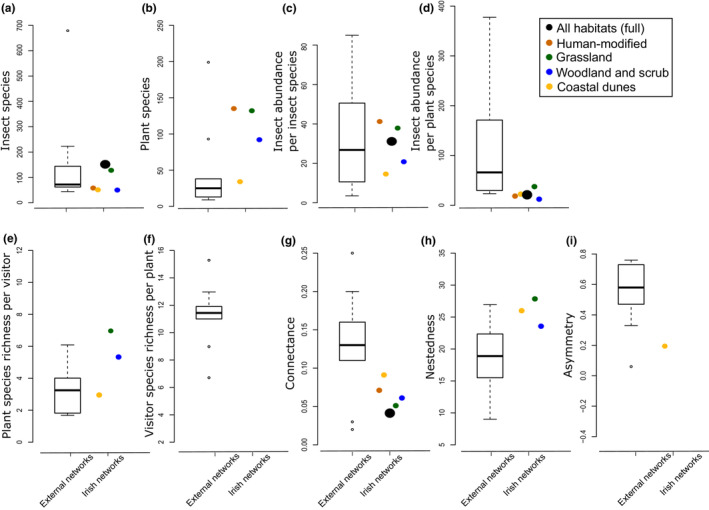
Box and whisker plots showing the network values of 10 published plant‐pollinator network studies (Table S2): insect species richness (a), plant species richness (b), insect abundance per insect species (c), insect abundance per plant species (d), plant species richness per visitor (e), visitor species richness per plant (f), connectance (g), nestedness (h), and asymmetry (i). Each plot contains the median (dark line), the upper and lower quartiles (box), the range (dotted lines), and various outliers (dots). The full Irish network (black) and four habitat types (intensively managed [primarily agriculture] = orange, coastal dunes = yellow, grasslands = green, and wood and shrublands = blue) are indicated by circles when they do not differ from the other published networks and stars when they differ significantly (by more than two standard deviations from the mean).

Relative to the other networks analyzed here, including those of comparable latitude, species richness, size, and habitat types, and including other islands, the full Irish network differed significantly (i.e., was more than two standard deviations from the mean) in the number of plant species, the average visitor richness per plant species, the average plant richness per visitor species, nestedness, and asymmetry (relative proportion of insect to plant species) (Figure 3, Table 3). The number of plant species surveyed in the full Irish network was higher than most of the other published networks we reviewed because this was a broader and more systematic country‐wide survey, including multiple habitat types, while the comparative studies were primarily conducted in a focal habitat type. However, the number of visitor species in the Irish network was not significantly different from the mean of the other networks. This resulted in the higher number of plant species per visitor, lower visitor species per plant, and overall negative asymmetry (more plant than insect species) in the Irish network. Though most flower‐visitor networks comprised, on average, 4.76 ± 0.74 times as many insect visitor species as flowering plant species, the Irish network comprised 1.61 times as many flowering plant species as visitor species. The Irish network also had a higher nestedness than the other published networks (Figure 3).

**TABLE 3:** Published datasets of plant‐insect visitor interactions from the Interaction Web Database and DataDryad and the network measures calculated for each of them

Country (publication)												
		Ireland	Canada (Barrett & Helenurm, 1987)	Sweden (Elberling & Olesen, 1999)	Seychelles (Kaiser‐Bunbury et al., 2017)	Japan (Kato et al., 1990)	UK (Memmott, 1999)	South Africa (Ollerton et al., 2003)	Germany, Sweden, UK (Magrach et al., 2018)	Spain (Bartomeus et al., 2008)	USA (Motten, 1986)	Argentina (Vázquez & Simberloff, 2002)
Duration (Years)		5	3	1	2	4	1	1	3	1	6	not reported
Latitude		52.98 N	46.56 N	68.21 N	−5.64	36.54	51.45	−30.08	51–56 N	42.3 N	35.77 N	−41
Hours surveyed		940	Not reported	230	1525	Not reported	Not reported	63	192	36	Not reported	452
Total Visits		4538	550	383	12,235	2459	2722	594	5973	1224	2225	5285
Habitat		Multiple	Boreal forest	Subarctic alpine	Mountaintop	Primary forest	Meadow	Upland grassland	Crop and grassland	Mediterranean shrublands	Deciduous forest	Evergreen montane forest
Sampling method		Transects	Plots	Transects	Transects	Transects	Transects	Plots	Transects	Transects	Plots	Plots
Plants sampled		All	12 focal species	All	All	All	All	9 focal species	All	All	13 focal species	All
Richness	*Pollinator*	148	102	118	144	679	79	56	223	81	44	90
	*Plant*	239	12	23	38	93	25	9	199	32	13	14
Weighted degree	*Pollinator*	30.66	5.39	3.25	84.97	3.52	27.63	10.61	26.78	15.15	50.57	58.72
	*Plant*	18.99	45.83	16.65	321.97	25.72	87.32	66	30.02	38.34	171.15	377.5
Unweighted degree	*Pollinator*	10.32	1.64	2.02	4.03	1.78	3.78	1.84	6.09	3.94	3.25	1.82
	*Plant*	6.39	13.92	10.35	15.29	12.97	11.96	11.44	8.98	9.97	11	11.71
Connectance		0.04	0.14	0.09	0.11	0.02	0.15	0.2	0.03	0.12	0.25	0.13
Nestedness		30.99	21.01	7.17	18.89	14.7	18.75	22.35	9.01	14.69	24.64	26.94
Asymmetry		−0.24	0.79	0.67	0.58	0.76	0.52	0.72	0.06	0.43	0.54	0.73

*Note:* Latitude is approximate for most studies, estimated according to the reported study area.

When comparing individual Irish habitats with the other published networks, we no longer saw a significant difference in the total number of plant species surveyed (Figure 3). However, in comparison to the published networks, Irish intensively managed habitats had a lower visitor richness per plant species, higher plant richness per visitor species, higher nestedness, and negative asymmetry. Irish coastal dunes differed only in that they had a lower visitor richness per plant species. Irish grasslands had a lower plant richness per visitor species and more negative asymmetry, and Irish woodland/shrubland habitats had fewer visitor species per plant species and a more negative asymmetry. When we divided the grassland into seminatural and managed habitats, the seminatural grasslands did not differ in any measure from the other published networks (Table 1). Thus, the difference between the grasslands overall and other networks were attributable to managed grasslands.

### 3.3 Relative species importance

**FIGURE 4:**
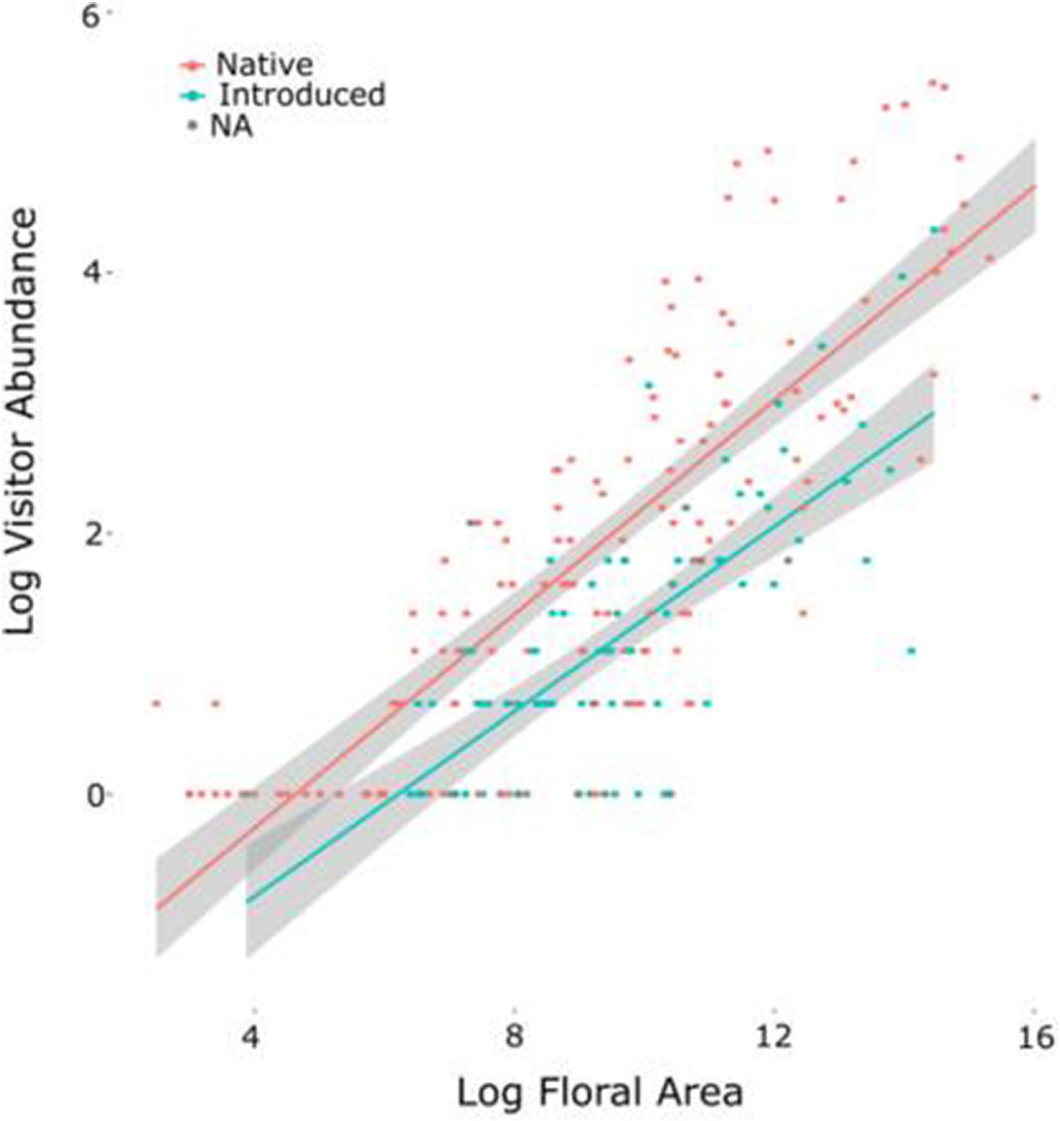
Scatterplot showing the significant relationship between log transformed floral area and log transformed average abundance of visitors in the full Irish network, with non‐native plant species (blue) significantly less attractive than native species (red). Gray points on the graph (NA) represent plant species where the origin is unknown, either because they are cultivars or because they are not resolved to the species level. The gray shaded area around the lines indicates the 95% confidence interval of a linear relationship.

Overall, the strongest predictor of insect visitation in the Irish plant–pollinator network was the size of the floral display (floral abundance × inflorescence size) (Figure 4). This was true both across all surveys (effect size 0.15, *z* value 20.76, *p* << .001) and across all plant species (effect size 0.39, *t* value 16.97, *p* << .001).

**FIGURE 5:**
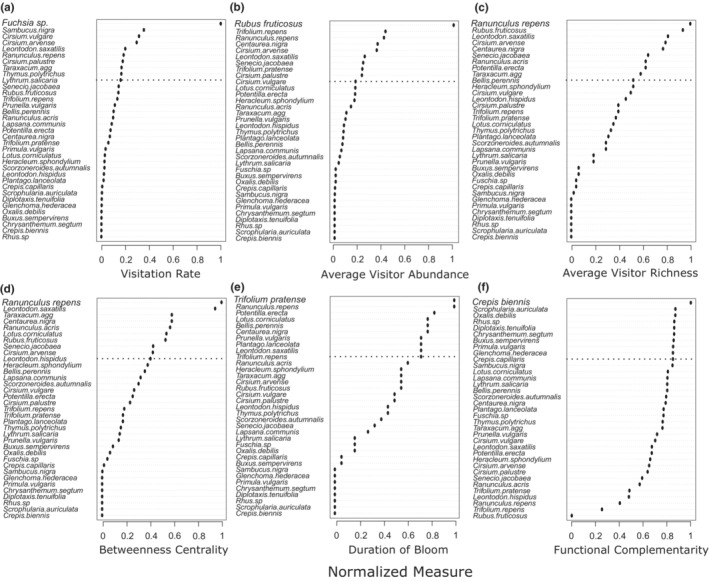
Dotplots showing the thirty‐five plant species in the Irish network that rank in the top ten of the different measures (visitation rate (a), average abundance of visitors (weighted degree, (b)), average species richness of visitors (unweighted degree, (c)), betweenness centrality (d), duration of bloom (node longevity, (e)), and functional complementarity (f)) ranked from highest (top) to lowest (bottom).

**FIGURE 6:**
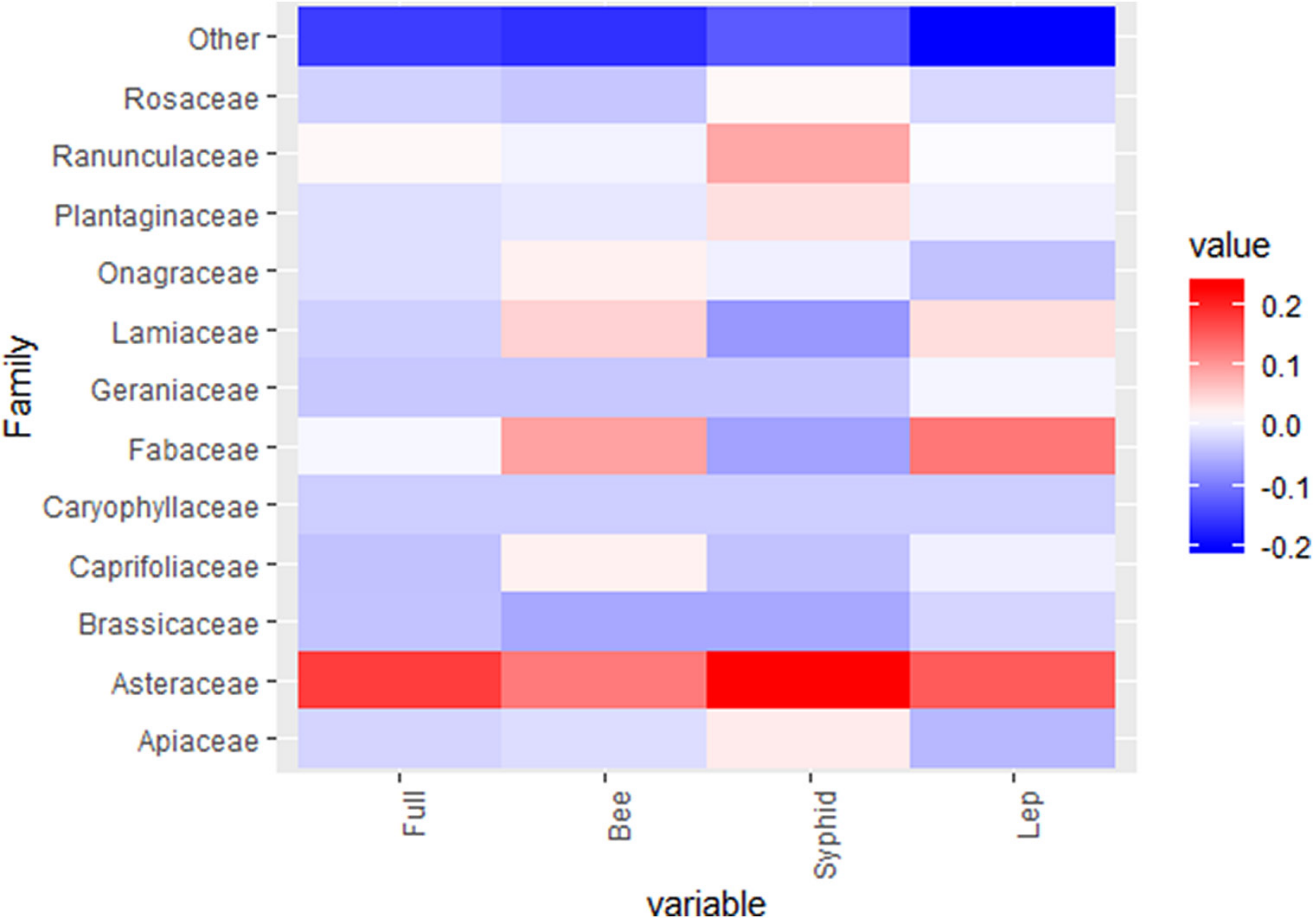
A heat map of the Irish network depicting the relative preferences of different insect visitor groups (all together (Full), bee, hoverfly (Syrphid), and moth and butterfly (Lep)). These demonstrate the prevalence of members of each plant family in the top ten rankings relative to the background floral abundance of plant species in each plant family in the overall network. Blue represents a group that is underrepresented in the top ten rankings relative to its background abundance, and red highlights groups that are overrepresented relative to their background abundance. The values are relative proportions different from background abundance. For example, syrphids prefer species of the family Asteraceae at 20% higher than the background abundance of these plant species in the network.

We ranked plant species in the network by eight measures, and then recorded the top ten species according to each metric. Thirty‐five plant species were ranked in the top ten of at least one measure (Figure 5). Four of these (11%) were non‐native, which was substantially lower than their prevalence in the overall network (31.7%), suggesting non‐native plant species were not preferred by insect visitors overall. Members of the family Asteraceae were particularly preferred, potentially because of their open floral morphology, although this varied among individual insect groups (Figure 6).

Bramble (*R. fruticosus* agg.) was found in all surveyed habitat types and had the highest average number of insect visitors, followed by *T. repens, Ranunculus repens* L.*, Centaurea nigra* L., and *Cirsium arvense* (L.) Scop. (Figure 5b). Creeping buttercup (*R. repens*) supported the highest average number of visitor species, followed by *R. fruticosus* agg., *Leontodon saxatilis* (Vill.) Mérat, *C. arvense*, and *C. nigra* (Figure 5c). *R. repens* was also visited by the largest number of insect species overall (49 of 148, or 33% of the insect species), and had the highest betweenness and closeness centrality (Figure 5d). Creeping buttercup (*R. repens*), also had the longest duration of bloom (highest node longevity), along with red clover (*Trifolium pratense* L.) (Figure 5e). The species with the highest functional complementarity were rough hawksbeard (*Crepis biennis* L.), followed by *Scrophularia auriculata* L.*, Oxalis debilis* Kunth*, Rhus* spp., and *Diplotaxis tenuifolia* (L.) DC. (Figure 5f). The non‐native *Fuchsia* cultivar had the highest visitation rate, followed by *Sambucus nigra* L.*, Cirsium vulgare* (Savi) Ten., *C. arvense*, and *L. saxatilis* (Figure 5a).

Among the distinct insect groups, *R. fruticosus* agg. supported the greatest abundance of syrphid and bee visitors, but *Lotus corniculatus* L. supported the greatest abundance of lepidopterans. Bees, moths, and butterflies also preferred the plant family Fabaceae over its background prevalence (Figure 6).

When ranking the insects, twenty‐eight insect species were ranked in the top ten of all metrics of “importance,” 53.6% of which were syrphid flies, 42.9% bees, and 3.6% Lepidoptera (Figure S5). While the proportion of syrphids in the top ten reflected their frequency in the database (54.7% syrphids), there were proportionally more bee species, but fewer Lepidoptera, ranked as highly important compared with their background prevalence (30.7% bee and 14.6% Lepidoptera).

When ranking the insect visitors, *Bombus pascuorum* Scopoli, ranked the highest for every measure except for functional complementarity, whereas *Halictus* Latreille ranked the highest (Figure S5). *B. pascuorum* visited 127 of the 239 plant species (53%).

Non‐native plant species represented 50% of plant species richness on average across surveys, but received only 11% of total visits. They also had a significantly lower average flower‐visitor abundance (effect size −0.83, *t* value −6.91, *p* < <.001, Figure 4). We found non‐native plant species differed significantly from native plant species in terms of their network roles (Table 4). They had a lower abundance and richness of visitors, bloomed for a shorter duration of the flowering season, and had a lower betweenness (but not closeness) centrality. Non‐native species did not differ from native species in their functional complementarity, visitation rate, or closeness centrality (Table 4).

**TABLE 4:** Results of generalized linear mixed effects models comparing network roles of native and non‐native plant species in the Irish network

Response	Fixed effect	Degrees freedom	Effect size	*t* Value	*p* Value
Visitation rate	Non‐native Y ‐ N	210	−1.97E‐04	−2.34	.02
**Average abundance of insects (weighted degree)**	**Non‐native Y ‐ N**	**210**	**−20.48**	**−2.80**	**.006**
**Average species richness of partners (unweighted degree)**	**Non‐native Y ‐ N**	**210**	**−4.68**	**−4.04**	**7.43E‐05**
Functional complementarity	Non‐native Y ‐ N	210	7.13	2.27	.02
**Duration of activity (node longevity)**	**Non‐native Y ‐ N**	**210**	**−2.16**	**−4.24**	**3.43E‐05**
**Betweenness centrality**	**Non‐native Y ‐ N**	**210**	**−31.71**	**−2.95**	**3.51E‐03**
Closeness centrality	Non‐native Y ‐ N	210	−1.50E‐04	−1.92	.056

*Note:* For these models, we set the network measure as the response variable, and the status of the plant (non‐native or native) as the fixed effect. The family for the models was Gaussian. We report the effect size, *t* value (Wald Statistic) and *p* value. To account for repeated tests, we use a Bonferroni correct alpha = 0.007. Significant effects at this alpha are highlighted and in bold.

## 4 DISCUSSION

As the intensity and extent of human land‐use increases (Tilman et al., 2011), it is important to consider how we can best protect communities of beneficial insects. More than half of Irish bee species have experienced significant declines in recent decades (Fitzpatrick et al., 2006). Clearly, the first step is to protect natural and seminatural habitats which retain beneficial biodiversity. In Irish landscapes, seminatural grassland habitats retained the highest proportion of unique species of both insect visitors and plant species: 40% of the visitor species in the grasslands were only found in that habitat and included four bee species listed as “vulnerable” by the recent red list of Irish bees (*Andrena angustior* Kirby, *A. semilaevis* Pérez, *Bombus ruderarius* Müller, and *Lasioglossum nitidiusculum* Curtis) (Fitzpatrick et al., 2006). There were only four plant species common to all habitat types, while insect species tended to be broadly distributed across habitats. The asymmetric distribution of insect versus plant species could be a result of sensitivity to different management practices, or related to the amount of habitat needed to sustain populations of plants vs. insects. Despite the fact that insect species are mobile, they appear to be more specialized in terms of habitat than their plant partners. This suggests that conserving habitats with unique insect species may be essential for pollinator conservation.

Our analysis also illustrated patterns in the phenology of the network, showing the highest abundance and species richness of both plant and visitor species in July, while the network was more connected in August. September had the highest nestedness and asymmetry of the months, but also was represented by relatively few plant and insect species. A study in Pennsylvania (USA), showed a similar pattern of the highest species richness in the middle of the summer, but also found the highest nestedness at that time, while connectance was highest early in the season (Russo et al., 2013). Interestingly, the full (year‐round) network was more negatively asymmetric than any month alone, because the plants tended to be active for shorter durations of the summer than their insect partners. This overlap in visitor communities among the months can also be illustrated by the overlap in their NMDS ordination.

Most flower‐visitor networks are highly asymmetric, with a much larger number of insect visitors than plant hosts. This asymmetry has implications for coevolutionary processes (Bascompte et al., 2006; Ramírez et al., 2011; Russo et al., 2019). In contrast, the compiled data for the Irish flower‐visitor network showed a strong asymmetry in the opposite direction: across all habitats, there were far more plant than visitor species. These differences were largely driven by intensively managed, especially agricultural, habitats in Ireland. Managed grasslands had the highest proportion of non‐native plant species, but did not have proportionally greater numbers of pollinator species. In fact, the managed grasslands are often deliberately seeded with agricultural species to increase their value for grazing or silage (Fossitt, 2000). Primarily agricultural habitats had few non‐native plants and relatively low pollinator species richness, suggesting non‐native plant species were not contributing to the network structure in that habitat. Woodland/shrubland habitats in Ireland also had negative asymmetry, possibly because the habitats in this category were dominated by samples from hedgerows, which tend to line agricultural fields, and non‐native shrubs, including ornamental plants found in nonagricultural intensively managed habitats. Seminatural grasslands had high flower‐visitor species richness and low proportions of non‐native plant species, leading to a positive asymmetry more in line with what was observed in networks outside Ireland.

The Irish network was also more nested than other networks, possibly because of the highly asymmetrical interactions, as nestedness has been linked to asymmetry (Bascompte et al., 2003). Moreover, it has been argued that nestedness relates to community persistence in mutualistic networks. On the other hand, the Irish network was significantly more nested than other networks we observed, and significant variation in nestedness among mutualistic networks calls into question its importance as a predictor of biodiversity. Moreover, nestedness in seed dispersal networks was higher in species‐rich networks that were located in areas of high human impact (Sebastián‐González et al., 2015) and may result from high environmental variability (Song et al., 2017).

Where habitat has been degraded, it is important to identify central plant species that support a large diversity and abundance of insects and those that support a unique complement of insect species, with high functional complementarity. If habitat loss due to land‐use change is causing a loss of pollinator species richness in Irish plant–insect visitor networks, it may be possible to reverse the effects by supplementing additional provisioning habitat containing native plant species (Menz et al., 2011). Indeed, managed grasslands were significantly different to seminatural grasslands, especially in terms of the number of non‐native plant species. In this case, because non‐native plant species were less attractive to flower‐visitors than native species, networks with large numbers of non‐native plant species had proportionally fewer pollinator species.

From the perspective of three groups of pollinating insects (bees, syrphid flies, and lepidopterans) (Russo et al., 2013), we provide evidence‐based recommendations for plants to be included in pollinator conservation in Ireland. This analysis showed cosmopolitan plant species with a broad distribution and high abundance of floral resources are central to the flower‐visitor network, and support the most abundant and widespread insect species. For example, if the objective is to maximize the abundance of insects, we recommend protecting species with a high average abundance of visitors (weighted degree). Across all flower‐visitors, bramble (*R. fruticosus* agg.) was found in all surveyed habitat types and had the highest average number of insect visitors, followed by *T. repens, Ranunculus repens* L.*, Centaurea nigra* L., and *Cirsium arvense* (L.) Scop. On the other hand, if the objective is to support a high diversity of flower‐visiting insects, we recommend protecting species with a high visitor species richness (unweighted degree). Creeping buttercup (*R. repens*) supported the highest average number of visitor species, followed by *R. fruticosus* agg., *Leontodon saxatilis* (Vill.) Mérat, *C. arvense*, and *C. nigra*. *R. repens* was also visited by the largest number of insect species overall (49 of 148, or 33% of the insect species), and had the highest betweenness and closeness centrality. Creeping buttercup (*R. repens*), also had the longest duration of bloom (highest node longevity), along with red clover (*Trifolium pratense* L.), and thus can provide resources throughout the flowering season. It is important to note, however, that the large role of these plant species in the community is likely due to their large distribution and high abundance. Thus, planting, or protecting, these species may have high value in supporting common and abundant flower‐visiting insects, but may not support uncommon or specialized insects. For this purpose, it may be wise to maximize functional complementarity. Here, the species with the highest functional complementarity were rough hawksbeard (*Crepis biennis* L.), followed by *Scrophularia auriculata* L.*, Oxalis debilis* Kunth*, Rhus* spp. (non‐native), and *Diplotaxis tenuifolia* (L.) DC. The plant species with high functional complementarity tended to be rarer, and also supported less common insect visitors. Although the non‐native *Fuchsia* cultivar had the highest visitation rate across all plant species, this effect was driven by a low number of records of this species in the database, where a low floral density (a single flower) in the sampled habitats was visited by a single bee. In contrast, for most plant species, hundreds or thousands of floral units were surveyed. After *Fuchsia*, the plants with the highest visitation rate were *Sambucus nigra* L.*, Cirsium vulgare* (Savi) Ten., *C. arvense*, and *L. saxatilis*.

The distinct preferences among the insect groups can also be applied to the overall rankings of the plants. The rankings of each of the other measures varied among the insect groups, and it is clear that conservation recommendations would vary depending on the insect group of interest. For example, bees preferred plant species in the Fabaceae family. Flowers from this family are typically zygomorphic and their morphology can restrict the insects capable of accessing pollen and/or nectar. Legume pollen is preferred forage by bumble bees in some systems, and can have a high protein content (Pywell et al., 2005).

The relative species richness of Syrphidae in the Irish network suggested they may be undervalued contributors to pollination services in Ireland. However, in terms of sheer abundance and number of plant species visited, the more abundant and generalized bees (especially *B. pascuorum* and *Bombus lucorum* agg.) out‐ranked Syrphidae and thus were more “important” by our measures. Similarly, to the plant species rankings, this is driven by the broad distribution and high abundance of these bumble bee species. These two common bumble bees were responsible for 30% of the total observed visits. In Ireland, highly generalized bumble bee species represent both the core of the network structure and also the vast majority of interactions.

Flower‐visiting insects provide a valuable ecosystem service and though pollination was not directly measured by this study, other studies have demonstrated the importance of flower‐visiting insect diversity to supply pollination services in agricultural systems (Dainese et al., 2019; Garibaldi et al., 2013). If there is a detrimental net effect of agriculture on flower‐visitor diversity, there are serious implications for the sustainability of pollination services for pollinator‐dependent crops and terrestrial ecosystems. Ireland has a long history of agriculture (Brown, 2007), and the highest proportion of land in Europe dedicated to agricultural production (Eurostat Agricultural Census, 2010), but the impact of agriculture is not restricted to Ireland. As the human population and demand on agricultural production increase, both the intensity and extent of agriculture can only increase globally (Tilman et al., 2011). Ireland may exemplify the future of plant–pollinator interactions globally. Declines in insect biomass have already been detected, even in protected areas (Hallmann et al., 2017; Lister & Garcia, 2018). Given the increase in pollinator‐dependent crop production, and the importance of food diversity for human health, biodiversity loss in pollinator communities is an unsustainable outcome. Identifying plant species with a central role in the broader flower‐visitor community, as we have done here, is an important first step in pollinator conservation.

## AUTHOR CONTRIBUTIONS

**Laura Russo:** Conceptualization (equal); data curation (equal); formal analysis (equal); funding acquisition (equal); investigation (equal); methodology (equal); validation (equal); visualization (equal); writing – original draft (equal); writing – review and editing (equal). **Úna Fitzpatrick:** Data curation (equal); investigation (equal); resources (equal); supervision (equal); validation (equal); writing – review and editing (equal). **Michelle Larkin:** Data curation (equal); investigation (equal); writing – review and editing (equal). **Sarah Mullen:** Data curation (equal); investigation (equal); validation (equal). **Eileen F Power:** Data curation (equal); funding acquisition (equal); investigation (equal); validation (equal); writing – review and editing (equal). **Dara Stanley:** Data curation (equal); funding acquisition (equal); investigation (equal); methodology (equal); validation (equal); writing – review and editing (equal). **Cian White:** Data curation (equal); investigation (equal); validation (equal); writing – review and editing (equal). **Jane Stout:** Conceptualization (equal); data curation (equal); funding acquisition (equal); investigation (equal); methodology (equal); project administration (equal); resources (equal); supervision (equal); validation (equal); writing – review and editing (equal).

## CONFLICT OF INTEREST

The authors have no conflict of interest.

## Supporting information


Appendix S1

## Data Availability

The data for this manuscript are available at DataDryad at the following DOI: https://doi.org/10.5061/dryad.kwh70rz47.
