# Supplementary material for: Conserving diversity in Irish plant–pollinator networks
Source: Ecol Evol. 2022 Oct 4;12(10):e9347. doi: 10.1002/ece3.9347 (PMC9532247; doi:10.1002/ece3.9347)
Supplement: Supplementary file 1 — Appendix S1 [file ECE3-12-e9347-s001.docx]

**Appendix for Conserving diversity in Irish plant-pollinator networks**

**Table S1.** Sampling effort in habitat subtypes, simplified to four main habitat types (B = human modified, C = coastal dunes, G = grasslands, W = wood and shrublands) where most of the sampling took place. Parts of this dataset were previously published to investigate the impact of agriculture on plant-pollinator interactions (Power and Stout 2011, Stanley and Stout 2013, Larking and Stanley 2021) and pollinator phenology (O’Rourke et al. 2014).

| **Habitat** | **Subcategory** | **Fossitt** (Fossitt 2000) **habitat categories** | **Area sampled (m^2^)** | **Time sampled (min)** | **Proportion non-native plant species** |
| --- | --- | --- | --- | --- | --- |
| **B** | ***BC1*** | Arable Crops | 1452 | 1345.63 | 0.09 |
| **B** | ***BC4*** | Flower beds and borders | 11023.87 | 686.06 | 0.49 |
| **B** | ***BL1*** | Stone walls and other stonework | 264 | 21 | 0.67 |
| **B** | ***BL2*** | Earth banks | 164 | 18 | 0 |
| **B** | ***BL3*** | Buildings and artificial surfaces | 1905.33 | 91 | 0.56 |
| **C** | ***CD2 & CD3*** | Marram dunes, Fixed dunes | 40000 | 400 | 0 |
| **C** | ***CD3*** | Fixed dunes | 62500 | 36378 | 0.03 |
| **G** | ***GA1 & GA2*** | Improved agricultural grassland, Amenity grassland | 22400 | 952 | 0.06 |
| **G** | ***GA1*** | Improved agricultural grassland | 62921.22 | 2694.58 | 0.06 |
| **GW** | ***GA1 & GS1 & WS1*** | Improved agricultural grassland, Dry calcareous and neutral grassland, Scrub | 105000 | 1050 | 0 |
| **G** | ***GA1 & GS1*** | Improved agricultural grassland, Dry calcareous and neutral grassland | 4200 | 150 | 0 |
| **G** | ***GA2*** | Amenity grassland (improved) | 2694.67 | 213.8 | 0.43 |
| **G** | ***GS1*** | Dry calcareous and neutral grassland | 130040 | 8183 | 0.03 |
| **G** | ***GS2*** | Dry meadows and grassy verges | 7189.72 | 533.83 | 0.10 |
| **GW** | ***GS1 & WS1*** | Dry calcareous and neutral grassland & Scrub | 245000 | 2450 | 0 |
| **W** | ***WD1*** | (Mixed) broadleaved woodland | 1264 | 105 | 0.38 |
| **W** | ***WD2*** | Mixed broadleaved/conifer woodland | 160 | 56 | 0 |
| **W** | ***WD5*** | Scattered trees and parkland | 1029.7 | 68.52 | 0.57 |
| **W** | ***WL1*** | Hedgerows | 4533.84 | 550.86 | 0.13 |
| **W** | ***WS3*** | Ornamental/non-native shrub | 5679.24 | 387.6 | 0.56 |
|  |  | **TOTAL** | **709721.59** | **56361.88** | **0.32** |

**Table S2.** Ecological interpretation of network properties measured in this study, chosen for their relationship with important structural attributes (McQuaid and Britton in press, Blüthgen et al. 2008, Vazquez et al. 2009, Martín González et al. 2010, Heleno et al. 2012), stability (Aizen et al. 2008, Thébault and Fontaine 2010), and robustness (Dunne et al. 2002, Campbell et al. 2012).

| **Network Properties** | **Alternative Name** | **Ecological Interpretation** |
| --- | --- | --- |
| **Unweighted Degree** | Species richness of partner | The number of species a given flower visitor or plant interacts with in the network. |
| **Weighted Degree** | Abundance of partner | The abundance of flower visitors, or flower visits, of species in the network. |
| **Connectance** | N/A | The connectance is the total number of interactions between species in the network divided by the number of possible interactions (number of plant species multiplied by number of insect species) (Heleno et al. 2012). |
| **Betweenness Centrality** | N/A | Calculated on an unweighted one-mode projection of the bipartite network for each group of species (insects and plants), this measures how central a species is by counting the number of “shortest paths” that must travel through the focal species. Centrality is a way of measuring the degree to which a species acts as a connector or hub between other groups of interacting species (Martín González et al. 2010). |
| **Closeness Centrality** | N/A | Calculated on an unweighted one-mode projection of the bipartite network for each group of species (insects and plants), this measures how central a species is by summing the length of its paths to all other species in the projection. A species with high centrality acts as a strong connector to other species (Martín González et al. 2010). |
| **Node Longevity** | Duration of activity | The number of weeks in the study season (May – September) that a species is active in the network. |
| **Functional Complementarity** | N/A | Measures the complementarity of species in the network with regard to their interaction profile. Species with lower complementarity are more similar to other species in the network, while species with high complementarity tend to support rare species or interactions. |
| **Nestedness (NODF)** | N/A | Quantifies to which degree interaction partners of specialized species are subsets of the partners of more generalized species (McQuaid and Britton in press). |
| **Asymmetry** | N/A | Measures the ratio in the number of plant and insect species interacting in the network. Positive values indicate more insect than plant species, while negative values indicate more plant than insect species. An asymmetry of 0 indicates equal numbers of plant and insect species. |
| **Network specialisation** | H_2_’ | Measures the mean specialisation of the network, across all species. This is calculated as H_2_’ (Blüthgen et al. 2006) and it describes to which extent the existing interactions are different than the expectations given marginal totals across the species. Higher network specialisation values indicate that the species in the network are more selective in their interaction partners. |
| **Specialisation asymmetry** | d' | Measures the asymmetry in the mean specialisation in insects vs. plants. Positive values indicate a higher specialisation of the insects. However, this specialisation asymmetry can be affected by the asymmetry in the number of species of the two interacting groups, so when there are far more plants than insects, the specialisation asymmetry is likely to be high (Blüthgen et al. 2006). |

**Table S3**. Insect visitor species common to all four habitat types, including the red list status of the bee species. (LC = least concern, NE = not evaluated, NT = near threatened)

| Group | Species | Bee Red List |
| --- | --- | --- |
| Bee | *Apis mellifera* | NE |
| Bee | *Bombus hortorum* | LC |
| Bee | *Bombus lapidarius* | NT |
| Bee | *Bombus lucorum* | LC |
| Bee | *Bombus lucorum agg* | NE |
| Bee | *Bombus muscorum* | NT |
| Bee | *Bombus pascuorum* | LC |
| Bee | *Bombus pratorum* | LC |
| Bee | *Bombus terrestris* | LC |
| Syrphid | *Cheilosia illustrata* |  |
| Syrphid | *Episyrphus balteatus* |  |
| Syrphid | *Eupeodes corollae* |  |
| Syrphid | *Eupeodes latifasciatus* |  |
| Syrphid | *Eupeodes luniger* |  |
| Bee | *Hylaeus communis* | LC |
| Bee | *Lasioglossum albipes* | LC |
| Lepidoptera | *Maniola jurtina* |  |
| Syrphid | *Melanostoma mellinum* |  |
| Syrphid | *Platycheirus albimanus* |  |
| Syrphid | *Platycheirus scutatus* |  |
| Syrphid | *Rhingia campestris* |  |
| Syrphid | *Sphaerophoria sp.* |  |

**Table S4.** Species unique to each of the four habitat types. Red list assignments based on Fitzpatrick et al (Fitzpatrick et al. 2006) and refer to the following: NE = Not Evaluated, NT = Near Threatened, LC = Least Concern, and VU = Vulnerable.

| **Group** | **Species** | **B** | **C** | **G** | **W** | **Bee Red List** |
| --- | --- | --- | --- | --- | --- | --- |
| Bee | *Nomada sp* | X |  |  |  |  |
| Bee | *Osmia bicornis* | X |  |  |  | NE |
| Lepidoptera | *Celastrina argiolus* | X |  |  |  |  |
| Lepidoptera | *Vanessa atalanta* | X |  |  |  |  |
| Syrphid | *Epistrophe elegans* | X |  |  |  |  |
| Syrphid | *Melanogaster hirtella* | X |  |  |  |  |
| Syrphid | *Neoascia geniculata* | X |  |  |  |  |
| Bee | *Andrena barbilabris* |  | X |  |  | NT |
| Bee | *Bombus monticola* |  | X |  |  | LC |
| Bee | *Colletes floralis* |  | X |  |  | VU |
| Bee | *Colletes similis* |  | X |  |  | NT |
| Bee | *Lasioglossum punctatissimum* |  | X |  |  | LC |
| Bee | *Osmia aurulenta* |  | X |  |  | NT |
| Lepidoptera | *Colias croceus* |  | X |  |  |  |
| Lepidoptera | *Macroglossum stellatarum* |  | X |  |  |  |
| Lepidoptera | *Zygaena lonicerae* |  | X |  |  |  |
| Syrphid | *Villa modesta* |  | X |  |  |  |
| Bee | *Andrena angustior* |  |  | X |  | VU |
| Bee | *Andrena bicolor* |  |  | X |  | LC |
| Bee | *Andrena cineraria* |  |  | X |  | LC |
| Bee | *Andrena lapponica* |  |  | X |  | LC |
| Bee | *Andrena minutula* |  |  | X |  | LC |
| Bee | *Andrena semilaevis* |  |  | X |  | VU |
| Bee | *Andrena wilkella* |  |  | X |  | DD |
| Bee | *Bombus ruderarius* |  |  | X |  | VU |
| Bee | *Bombus sylvestris* |  |  | X |  | LC |
| Bee | *Halictus rubicundus* |  |  | X |  | LC |
| Bee | *Halicutus sp* |  |  | X |  |  |
| Bee | *Lasioglossum* |  |  | X |  |  |
| Bee | *Lasioglossum fratellum* |  |  | X |  | LC |
| Bee | *Lasioglossum nitidiusculum* |  |  | X |  | VU |
| Bee | *Lasioglossum villosulum* |  |  | X |  | LC |
| Bee | *Nomada ruficornis* |  |  | X |  |  |
| Bee | *Sphecodes monilicornis* |  |  | X |  | LC |
| Lepidoptera | *Aglais polychloros* |  |  | X |  |  |
| Lepidoptera | *Hipparchia semele* |  |  | X |  |  |
| Lepidoptera | *Inachis io* |  |  | X |  |  |
| Lepidoptera | *Leptidea sinapis* |  |  | X |  |  |
| Lepidoptera | *Lycaena phlaeas* |  |  | X |  |  |
| Lepidoptera | *Zygaena filipendulae* |  |  | X |  |  |
| Syrphid | *Anasimyia lineata* |  |  | X |  |  |
| Syrphid | *Cheilosia albitarsis* |  |  | X |  |  |
| Syrphid | *Cheilosia latifrons* |  |  | X |  |  |
| Syrphid | *Cheilosia pagana* |  |  | X |  |  |
| Syrphid | *Cheilosia sp* |  |  | X |  |  |
| Syrphid | *Cheilosia vernalis* |  |  | X |  |  |
| Syrphid | *Chrysogaster* |  |  | X |  |  |
| Syrphid | *Chrysotoxum bicinctum* |  |  | X |  |  |
| Syrphid | *Epistrophe grossulariae* |  |  | X |  |  |
| Syrphid | *Eristalis horticola* |  |  | X |  |  |
| Syrphid | *Eristalis interruptus* |  |  | X |  |  |
| Syrphid | *Eristalis intricaria* |  |  | X |  |  |
| Syrphid | *Eupeodes bucculatus agg* |  |  | X |  |  |
| Syrphid | *Ferdinandea cuprea* |  |  | X |  |  |
| Syrphid | *Helophilus hybridus* |  |  | X |  |  |
| Syrphid | *Lejogaster metallina* |  |  | X |  |  |
| Syrphid | *Leucozona* |  |  | X |  |  |
| Syrphid | *Neoascia* |  |  | X |  |  |
| Syrphid | *Parasyrphus nigritarsus* |  |  | X |  |  |
| Syrphid | *Pipiza noctiluca* |  |  | X |  |  |
| Syrphid | *Platycheirus* |  |  | X |  |  |
| Syrphid | *Platycheirus angustatus* |  |  | X |  |  |
| Syrphid | *Platycheirus clypeatus* |  |  | X |  |  |
| Syrphid | *Platycheirus melanopsis* |  |  | X |  |  |
| Syrphid | *Platycheirus ramsarensis* |  |  | X |  |  |
| Syrphid | *Platycheirus rosarum* |  |  | X |  |  |
| Syrphid | *Platycheirus scambus* |  |  | X |  |  |
| Syrphid | *Platycheirus sticticus* |  |  | X |  |  |
| Syrphid | *Platycheirus tarsalis* |  |  | X |  |  |
| Syrphid | *Pyrophaena* |  |  | X |  |  |
| Syrphid | *Scaeva selenitica* |  |  | X |  |  |
| Syrphid | *Sphaerophoria interrupta* |  |  | X |  |  |
| Syrphid | *Xanthogramma citrofasciatus* |  |  | X |  |  |
| Bee | *Megachile centuncularis* |  |  |  | X | NT |
| Bee | *Megachile sp* |  |  |  | X |  |
| Bee | *Megachile versicolor* |  |  |  | X | LC |
| Lepidoptera | *Anthocharis cardamines* |  |  |  | X |  |
| Syrphid | *Baccha elongata* |  |  |  | X |  |
| Syrphid | *Eumerus strigatus* |  |  |  | X |  |

**Table S5**. We checked the datasets in relation to taxonomy, and eliminated groups other than syrphid flies, bees, and lepidoptera where the identity of the visitors was reported. In this repeated analysis, we excluded the Elberling & Olesen (1999) dataset as it was comprised of primarily non-syrphid diptera. Our main results did not differ significantly following this repeated analysis.

| **Country** |  | **Ireland** | **Canada** | **Seychelles** | **Japan** | **UK** | **South Africa** | **Germany, Sweden, UK** | **Spain** | **USA** | **Argentina** |
| --- | --- | --- | --- | --- | --- | --- | --- | --- | --- | --- | --- |
| **Years** |  | 5 | 3 | 2 | 4 | 1 | 1 | 3 | 1 | 6 | not reported |
| **Lat** |  | 52.98 N | 46.56 N | -5.64 | 36.54 | 51.45 | -30.08 | 51 - 56 N | 42.3 N | 35.77 N | -41 |
| **Hours** |  | 940 | not reported | 1525 | not reported | not reported | 63 | 192 | 36 | not reported | 452 |
| **Total Visits** |  | 4538 | 550 | 12235 | 2459 | 2722 | 594 | 5973 | 1224 | 2225 | 5285 |
| **Habitat** |  | multiple | boreal forest | mountaintop | primary forest | meadow | upland grassland | crop and grassland | meditteranean shrublands | deciduous forest | evergreen montane forest |
| **Richness** | *pollinator* | 148 | 72 | 144 | 679 | 70 | 56 | 223 | 62 | 44 | 90 |
|  | *plant* | 239 | 11 | 38 | 93 | 25 | 9 | 199 | 31 | 13 | 14 |
| **Weighted degree** | *pollinator* | 30.66 | 4.17 | 84.97 | 3.52 | 30.74 | 10.61 | 26.78 | 11.68 | 50.57 | 58.72 |
|  | *plant* | 18.99 | 29.55 | 321.97 | 25.72 | 86.08 | 66 | 30.02 | 23.35 | 171.15 | 377.5 |
| **Unweighted degree** | *pollinator* | 10.32 | 1.68 | 4.03 | 1.78 | 4.01 | 1.84 | 6.09 | 3.4 | 3.25 | 1.82 |
|  | *plant* | 6.39 | 11.91 | 15.29 | 12.97 | 11.24 | 11.44 | 8.98 | 6.71 | 11 | 11.71 |
| **Connectance** |  | 0.04 | 0.15 | 0.11 | 0.02 | 0.16 | 0.2 | 0.03 | 0.11 | 0.25 | 0.13 |
| **Nestedness** |  | 30.99 | 15.96 | 18.89 | 14.7 | 21.59 | 22.35 | 9.01 | 15.5 | 24.64 | 26.94 |
| **Asymmetry** |  | -0.24 | 0.73 | 0.58 | 0.76 | 0.47 | 0.72 | 0.06 | 0.33 | 0.54 | 0.73 |

**Table S6.** Pearson correlation matrix of the measured network properties. Significant (P < 0.05) correlations of r > 0.5 or r < -0.5 are highlighted in bold.

|  | **Weighted Degree** | **Unweighted Degree** | **Floral Area** | **Node Longevity** | **Visitation Rate** | **Betweenness Centrality** | **Closeness Centrality** |
| --- | --- | --- | --- | --- | --- | --- | --- |
| **Functional Complementarity** | **-0.75** | **-0.63** | -0.35 | **-0.56** | -0.23 | **-0.53** | -0.44 |
| **Weighted Degree** |  | **0.82** | 0.45 | **0.66** | 0.3 | **0.69** | **0.48** |
| **Unweighted Degree** |  |  | **0.5** | **0.85** | 0.37 | **0.92** | **0.66** |
| **Floral Area** |  |  |  | 0.33 | 0.09 | 0.44 | 0.36 |
| **Node Longevity** |  |  |  |  | 0.34 | **0.8** | **0.66** |
| **Visitation Rate** |  |  |  |  |  | 0.31 | 0.26 |
| **Betweenness Centrality** |  |  |  |  |  |  | **0.71** |

**Figure S1**


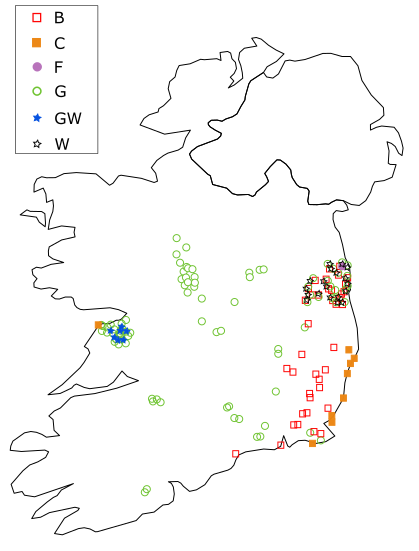


**Figure S1.** Map of surveyed sites in Ireland, with 119 survey sites in different habitat types varying in colour and shape. Cultivated land (B) = red, open square; coastal dunes (C) = orange, closed square; tall-herb swamps (F) = purple, closed circle; semi-natural grasslands (G) = green, open circle; mixed woodland and grassland (GW) = blue, closed star; woodland (W) = black, open star.

**Figure S2.**


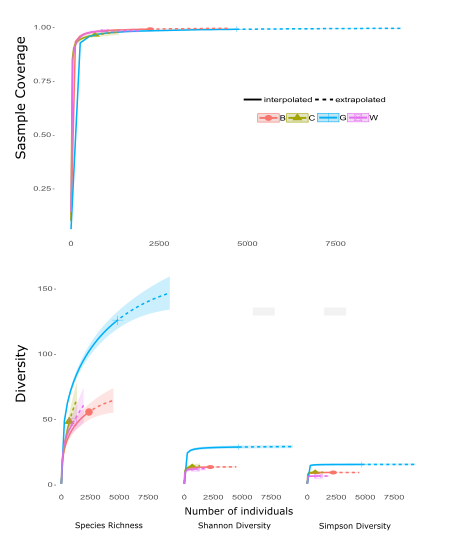


**Figure S2.** Species accumulation curves and rarefaction analysis (Chao et al. 2014) for the four different habitat types. Sample coverage varies from 96 – 99% in the species accumulation curve. The rarefaction analysis demonstrates a significantly higher species richness, Simpson’s, and Shannon’s diversity of flower-visiting insects in grassland habitats.

**Figure S3.**
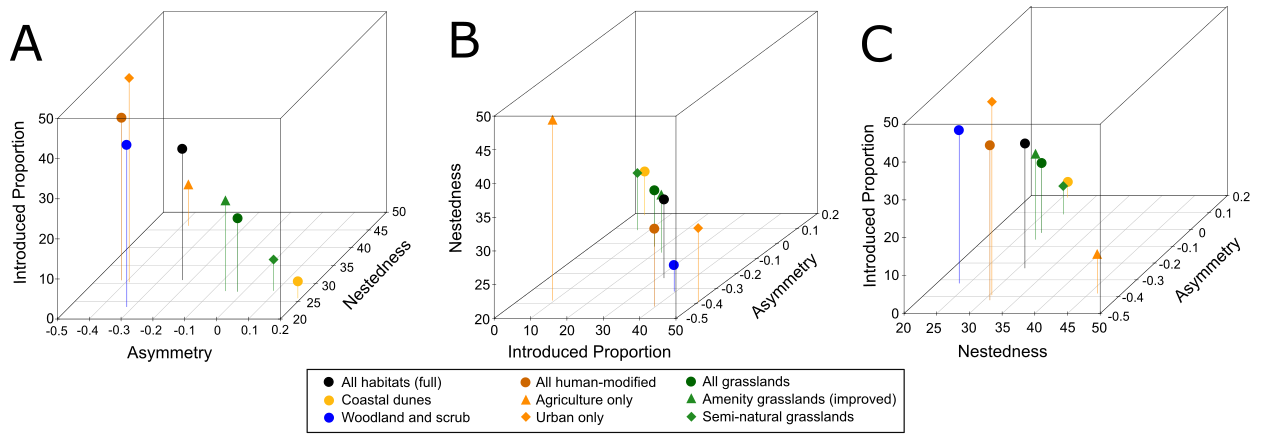


**Figure S3.** Graphs showing the relationship between network asymmetry, nestedness, and proportion of introduced plant species for the habitat types, with the human-modified habitats (orange circle) separated into urban (orange diamond) and agricultural (orange triangle) and grasslands (green circle) separated into semi-natural (green diamond) and improved (for agriculture) grasslands (green triangle). There is an association between the proportion of introduced plant species and asymmetry (plant species richness inflation) for most habitat types except for the agricultural lands, which have a low proportion of introduced plant species, but still a strong negative asymmetry (defaunation). The agricultural habitats (orange triangle) also have high nestedness.

**Figure S4.**


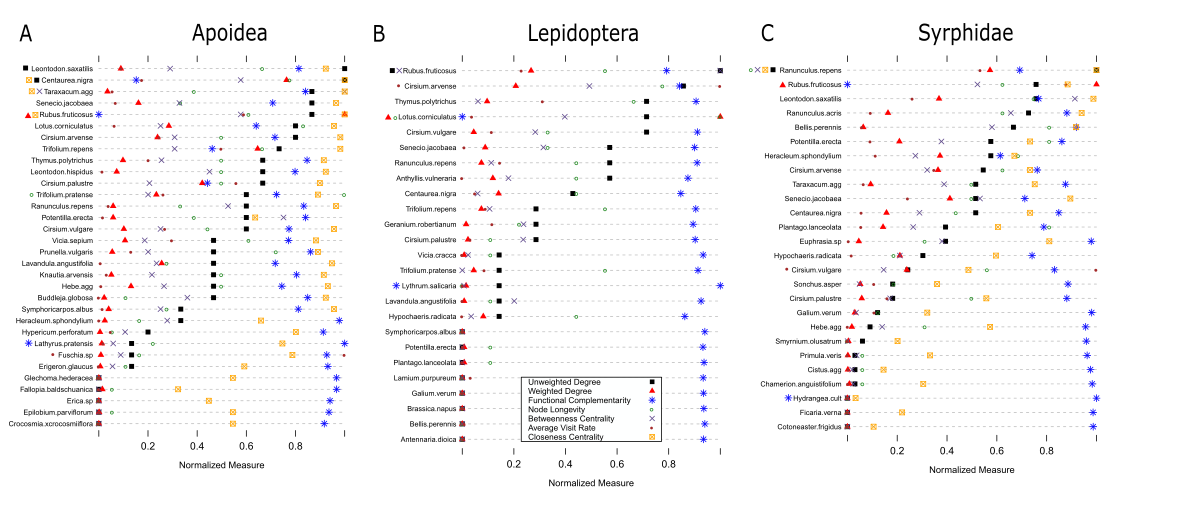


**Figure S4**. Dot plot rankings using normalized (feature scaled) measures for the plant species in the Irish network as ranked by the separate insect groups, Apoidea (bees), Lepidoptera (moths and butterflies), and Syrphidae (hoverflies). The species are ranked by highest unweighted degree from top to bottom, and other measured attributes are marked by symbols. The species with the highest value for each measure is marked with a symbol to the left of its name. Each group exhibits distinct preferences for different plant species.

**Figure S5**.


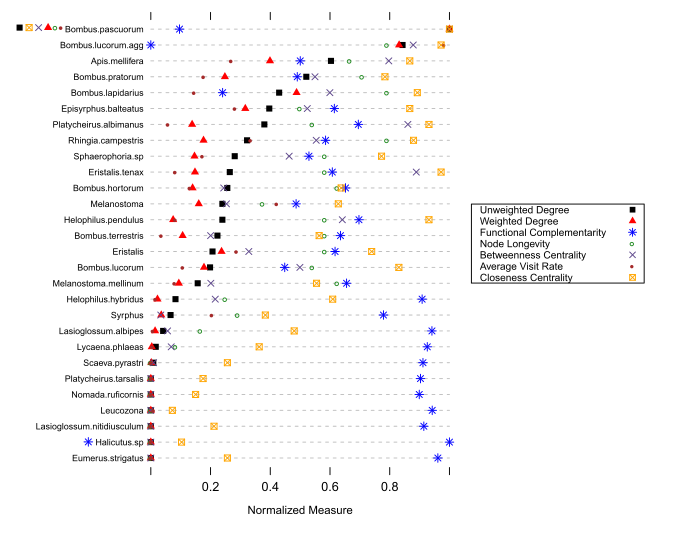


**Figure S5.** Dot plot rankings of the insect visitor species in the Irish network, using normalized (feature scaled) measures. The species are ranked by highest to lowest unweighted degree from top to bottom. *Bombus pascuorum* (the common carder bee), ranks the highest among most of the measures, except functional complementarity, where bees of the genus *Halictus* rank the highest.

**Figure S6.**


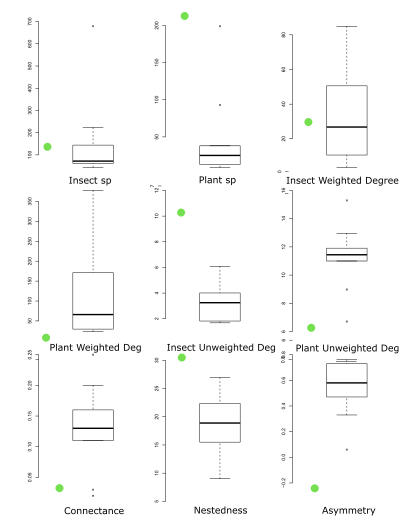


**Figure S6.** The Irish network measures (green circle) relative to the measures of the other published networks where we excluded a dataset with only non-syrphid flies and restricted the taxa of the networks to syrphids, bees, and Lepidoptera (where taxonomy was reported). This exclusion did not change our main results and the Irish network still stands out as having the lowest asymmetry and plant weighted average degree, and the highest nestedness, plant species richness, and insect unweighted average degree.
